# Impact of Inductively Coupled Plasma Etching Conditions on the Formation of Semi-Polar ($11\bar{2}2$) and Non-Polar ($11\bar{2}0$) GaN Nanorods

**DOI:** 10.3390/nano10122562

**Published:** 2020-12-20

**Authors:** Pierre-Marie Coulon, Peng Feng, Tao Wang, Philip A. Shields

**Affiliations:** 1 Department of Electrical & Electronic Engineering, University of Bath, Bath BA2 7AY, UK; ps229@bath.ac.uk; 2 Department of Electronic and Electrical Engineering, University of Sheffield, Sheffield S1 4DE, UK; pfeng3@sheffield.ac.uk (P.F.); t.wang@sheffield.ac.uk (T.W.)

**Keywords:** GaN, inductively coupled plasma, dry etching, nanostructures, morphology, light emitting devices

## Abstract

The formation of gallium nitride (GaN) semi-polar and non-polar nanostructures is of importance for improving light extraction/absorption of optoelectronic devices, creating optical resonant cavities or reducing the defect density. However, very limited studies of nanotexturing via dry etching have been performed, in comparison to wet etching. In this paper, we investigate the formation and morphology of semi-polar ($11\bar{2}2$) and non-polar ($11\bar{2}0$) GaN nanorods using inductively coupled plasma (ICP) etching. The impact of gas chemistry, pressure, temperature, radio-frequency (RF) and ICP power and time are explored. A dominant chemical component is found to have a significant impact on the morphology, being impacted by the polarity of the planes. In contrast, increasing the physical component enables the impact of crystal orientation to be minimized to achieve a circular nanorod profile with inclined sidewalls. These conditions were obtained for a small percentage of chlorine (Cl_2_) within the Cl_2_ + argon (Ar) plasma combined with a low pressure. Damage to the crystal was reduced by lowering the direct current (DC) bias through a reduction of the RF power and an increase of the ICP power.

## 1. Introduction

Semi-polar and non-polar gallium nitride (GaN) crystal orientations have received considerable attention owing to their many potential advantages over the polar orientation of c-plane GaN. They offer the possibility to reduce or eliminate polarization-related effects, such as increasing the radiative recombination rate, and mitigating the quantum-confined Stark effect, whilst also providing solutions to efficiency droop and the green gap, two challenging issues of current III-nitride light-emitting diodes (LEDs) grown from c-plane GaN [1,2].

So far, some remarkable external quantum efficiency (EQE) values have been reported across the purple [3], the blue [4,5,6] and the green wavelength ranges [7,8]. Yet, among these results, and many more showing reasonable performances, all were obtained from freestanding semi-/non-polar GaN substrates [1,2], while only a few mentioned the use of surface patterning to increase light extraction [4,5]. Pan et al. demonstrated the first EQE >50% [5] which is still one of the best values to date. However, to satisfy industrial requirements, semi-/non-polar LEDs need to be grown on low cost ≥2-inch foreign substrates, such as sapphire or silicon, instead of freestanding semi-/non-polar GaN with their small size and high preparation costs. In addition, optimization in light extraction efficiency must be carried out to further improve the device performances. As such, although promising, semi-/non-polar-GaN-based LEDs are inevitably facing two challenges already encountered in c-plane GaN: the large density of defects arising from epitaxial growth on foreign substrates and the limitation of light extraction due to total internal reflection resulting from the large refractive index difference between GaN and air.

Several approaches have been developed to reduce the defect density [9], with a particular focus on basal-plane stacking faults (BSFs). Indeed, while the stacking fault plane is commonly perpendicular to the growth direction in c-plane GaN, it is inclined or parallel to the growth direction in semi- and non-polar GaN, therefore directly impacting the electrical and optical performance of the devices. A solution to avoid the propagation of defects such as BSFs or threading dislocations involves first, the use of substrate patterning or GaN defect filtering strategies (e.g., via masking of the GaN surface or top-down removal of GaN), and second, the overgrowth of GaN, which can proceed in multiple steps in order to overlie the defective region [9].

As for improving light extraction, while c-plane GaN surface texturing has been widely demonstrated either on the p-GaN top or n-side-up GaN surfaces via wet etching [10,11] and dry etching [12,13], literature on surface texturing of semi-/non-polar GaN is sparse, with few reports on wet etching [14,15,16], and even fewer on dry etching [17]. Although promising, wet etching systematically leads to the formation of highly inhomogeneous trigonal prisms having one (0001) facet and two {$10\bar{1}0$} m-plane facets, and this regardless of the semi- or non-polar GaN crystal orientation.

The fabrication of highly organized and uniform textured surfaces with tuneable profile and etch depth is therefore of high interest to reduce the defect density and improve light extraction. Furthermore, it could also be beneficial for the creation of resonant nano-microcavites. In this work, we combine an emerging nano-patterning technique called Displacement Talbot lithography (DTL) with top-down dry etching to investigate the impact of inductively coupled plasma (ICP) conditions on the formation of nanorod structures from ($11\bar{2}2$) semi-polar and ($11\bar{2}0$) non-polar GaN layers. Parameters such as the chemistry, the pressure, the temperature, the radio-frequency (RF) and ICP power and time are explored. The morphology of the nanorods and their characteristics, such as the circularity, the etch depth, and the sidewall profile were characterized by scanning electron microscopy (SEM) after etching. From this, we deduce that a small percentage of chlorine (Cl_2_) into the Cl_2_ + Ar plasma combined with a low pressure enables achieving a circular nanorod profile with inclined sidewalls independently of the crystal orientation.

## 2. Materials and Methods

**Figure 1:**
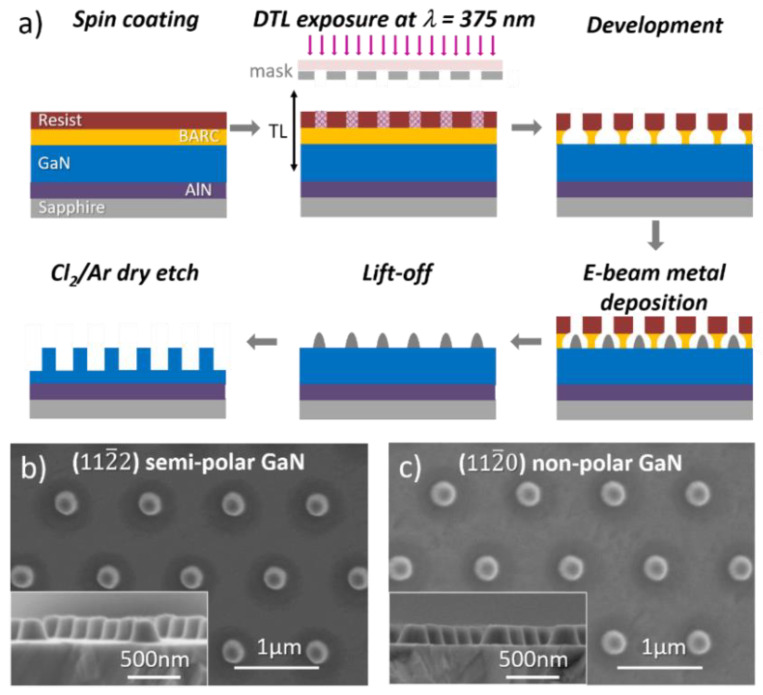
(**a**) Sketch of the process flow for fabrication of gallium nitride (GaN) nanorods. Plan view and cross-sectional (inset) scanning electron microscopy (SEM) image of nickel mask on (**b**) ($11\bar{2}2$) semi-polar and (**c**) ($11\bar{2}0$) non-polar GaN layers.

Semi-polar ($11\bar{2}2$) and non-polar ($11\bar{2}0$) GaN layers were grown by metal organic vapor phase epitaxy respectively on 2-inch m- and r-plane sapphire. The two epitaxial structures respectively consisted of 220 and 230 nm AlN buffer layers followed by 1.3 and 1.2 µm GaN. Figure 1a presents the use of a DTL-based (PhableR 100, Eulitha, Kirchdorf, Switzerland) [18] lift-off technique to create highly regular hexagonal arrays of nickel nanodots to be used as a hard mask for nanorod etching experiments [19,20]. The wafers were spin coated with a ~270 nm bottom anti reflective coating (BARC) layer (WiDE^®^ 30W-Brewer Science, Rolla, MO, USA) and a ~360 nm high-contrast positive resist layer (Dow^®^ Ultra-i 123 diluted with Dow^®^ EC11 solvent). A 1 µm pitch hexagonal array of nanoholes was then formed by DTL. The undercut profile created in the BARC (cured at 150 °C) after exposure and development was used as a lift-off layer. Metal was then deposited via e-beam evaporation and lift-off was achieved by soaking the wafer in the developer. The resulting array of metal nanodots (Figure 1b,c) was transferred into the GaN layer via an ICP dry etch system (Oxford Instruments System 100 Cobra, Bristol, United Kingdom). A different series of experiments, in which only one parameter was varied, were performed in order to study the influence of the Cl_2_/Ar flow rate, chamber pressure and temperature (electrode temperature at 20 °C was controlled by a fluid re-circulating chiller and higher electrode temperatures were controlled by a resistance heater), RF and ICP power and time on the morphology and characteristics of etched nanorod arrays, as summarized in Table 1. The characteristics of etched nanorods are the following: (1) the etch rate, corresponding to the measured height of the nanorod divided by the time in nm/min, (2) the pedestal diameter, being the diameter extracted at the bottom of the nanorod from plan-view SEM images, and (3) the sidewall angle, defined as the angle between the nanorod sidewall and an “ideal” vertical straight sidewall and estimated from cross-sectional SEM images. In addition to ($11\bar{2}2$) semi-polar and ($11\bar{2}0$) non-polar GaN layers, a commercial ~7 µm c-plane GaN template was also used in the investigation for comparison. The size of each GaN sample was around 1 × 1 cm^2^.

## 3. Results and Discussion

### 3.1. Effect of Chemistry: Cl_2_/Ar

**Figure 2:**
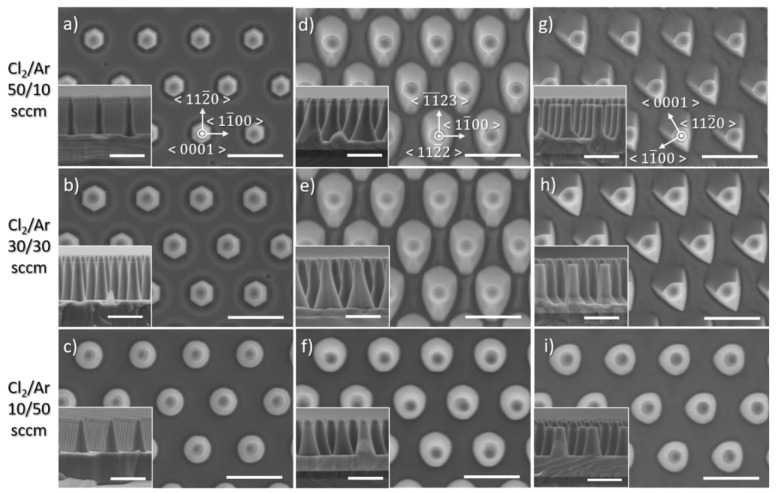
Plan view and cross-sectional (inset) SEM image of GaN nanorod etch series 1 with different ratios of Cl_2_/Ar flow rates for (**a**–**c**) *c*-plane (**d**–**f**) ($11\bar{2}2$) semi-polar and (**g**–**i**) ($11\bar{2}0$) non-polar GaN layers. Scale bar is 1 µm.

Figure 2 shows SEM images of GaN nanorods etched from three GaN crystal orientations: the polar *c*-plane (Figure 2a–c), the ($11\bar{2}2$) semi-polar plane (Figure 2d–f) and the ($11\bar{2}0$) non-polar plane (Figure 2g–i), from the left to right. All were etched for three different Cl_2_/Ar ratios, with the value on the left corresponding to that row. All plan-view SEM images have been acquired along the same orientation which is displayed in the upper row for the three different templates. In contrast, the cross-section SEM images have not been acquired along the same orientation as it is practically more complicated to cleave each sample along the same direction. Note that the configuration and description of the SEM images given above has been systematically employed for the various etch series.

**Figure 3:**
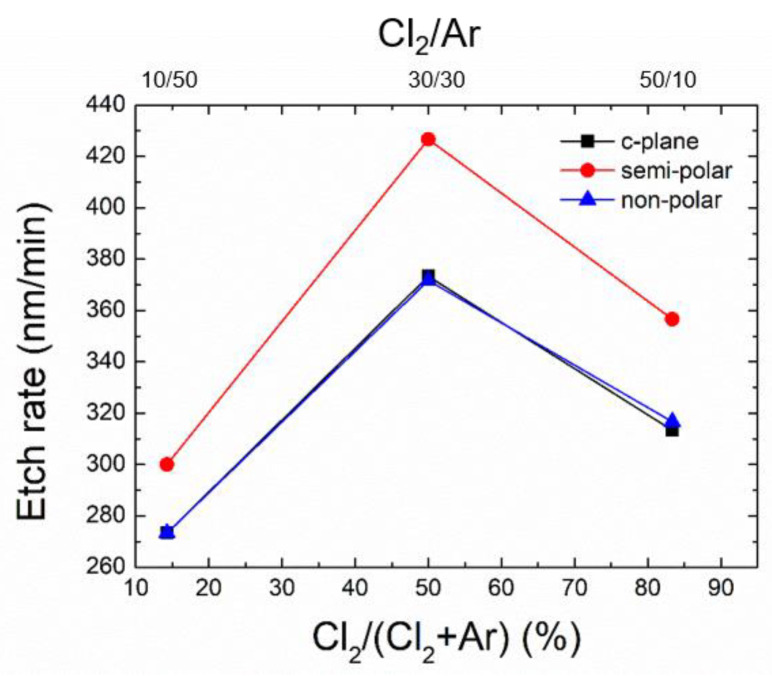
Etch rate of the *c*-plane, ($11\bar{2}2$) semi-polar and ($11\bar{2}0$) the non-polar GaN layers as a function of the Cl_2_/Ar ratio.

For 50 sccm Cl_2_ and 10 sccm argon (Ar) (upper row), the morphology of the nanorod is found to be highly dependent on the crystal orientation, with a fairly straight nanorod profile for the polar *c*-plane (Figure 2a), a tapered profile for the ($11\bar{2}2$) semi-polar plane (Figure 2d), with various sidewall angles depending on the position near the top or lower part of the nanorod, and, what seems to be a mixed straight and tapered profile for the ($11\bar{2}0$) non-polar plane (Figure 2g), again different in the upper part and lower part of the nanorod. Similarities are also observed between the different crystal orientations, with the observation of a faceting, being clearly hexagonal for the polar *c*-plane and conical or triangular for the ($11\bar{2}2$) semi-polar and ($11\bar{2}0$) non-polar planes. Noticeably, the sidewall angle along the {$1\bar{1}00$} direction appears to be the smallest for all three crystal orientations. For equal flows of Cl_2_ and Ar for the same overall flow rate (middle row), the results for the three crystal orientations are similar, except for the base of the nanorod being larger for all crystal orientations, which is most likely due to a greater etch depth (see etch rate in Figure 3). Finally, when the proportion of Cl_2_ is further decreased, with 10 sccm Cl_2_ and 50 sccm Ar (lower row), no clear facets are formed; instead the base of the nanorod is circular for the polar *c*-plane (Figure 2c), which is then slightly distorted for the ($11\bar{2}2$) semi-polar (Figure 2f) and ($11\bar{2}0$) non-polar templates (Figure 2i). The tapering profile is now more or less uniform, in contrast to the two previous etching conditions. Note that experiments were attempted for Cl_2_ flow rates below 10 sccm and Ar flow rates above 50 sccm to further reduce the proportion of Cl_2_ in the plasma, however, without any success as the strong fluctuations in the RF and ICP reflected power resulted in an unstable plasma.

The etch rate of GaN for the three crystal orientations has been extracted from SEM cross-section images and is given in Figure 3 as a function of the Cl_2_/Ar ratio or the percentage of Cl_2_ to the Cl_2_ + Ar plasma, for a constant total flow of 60 sccm. Independently of the orientation, the higher and lower etch rate are respectively observed at 50% (30/30) and 14% (10/50), while irrespective of the Cl_2_/Ar ratio, the etch rate is found to be higher for ($11\bar{2}2$) the semi-polar plane over *c*- and ($11\bar{2}0$) the non-polar planes.

In an ICP GaN Cl_2_/Ar based etching process, the physical component of etching arises from active ions bombarding the surface (Ar^+^, Cl^+^, and Cl_2_^+^ as well as SiCl^+^ and SiCl_3_^+^ coming from the Si carrier wafer employed for these experiments) and the chemical one originates from dissociated radicals (Cl) and their interaction with the surface. While the energetic ion bombardment sputters the surface and breaks the strong GaN chemical bonds, the Cl radicals are absorbed on the dangling bonds (DB) of Ga atoms, creating a chemical reaction associated with the formation of volatile etched by-products (GaCl_x_ and N_2_). As Cl_2_ is substituted by Ar, the Cl radical density will gradually decrease while the ion current density will increase [21], meaning that the chemical etching component is progressively reduced in favour of the physical one.

Therefore, the high etch rate observed at 30/30 sccm (50%) means that ion bombardment accelerates both the Cl radical reaction with the surface and the etch by-product desorption. In contrast, at 10/50 sccm (14%) the Cl radical reaction with the surface is limited compared to the etch by-product desorption and vice versa at 50/10 sccm (83%). Although not exactly identified in this etch series, the maximum etch rate will be achieved at specific plasma compositions, for an optimal neutral-to-ion ratio that provides enough Cl radical surface coverage and reaction, and subsequent etch by-product desorption [22].

For the different plane orientations, a change in the ratio of Ga to N atoms at the GaN surface is to be expected, as well as for the density of DBs per unit area (or DB per Ga atom). If we consider the three orientations investigated here, the (0001) plane is Ga terminated with a DB density of 11.4 nm^−2^ (one DB per Ga atom), the ($11\bar{2}2$) plane is Ga terminated with a DB density of 17.8 nm^−2^ (two DBs per Ga atom) and the ($11\bar{2}0$) plane contains an equal number of Ga and N atoms with DB density of 14.0 nm^−2^ (one DB per Ga atom). As such, the ($11\bar{2}2$) plane is more likely to promote the generation of GaCl_x_ compared to the two other orientations, which corroborates the higher etch rate of the ($11\bar{2}2$) plane compared to *c*- and the ($11\bar{2}0$) non-polar planes. This difference in the etch rate however changes as a function of the Cl_2_/Ar ratio, and thus the related formation of GaCl_x_, with the difference being large (~50–60 nm/min) when chemical etching is more dominant, and smaller (~30 nm/min) when physical etching is more dominant.

Finally, the variation in the nanorod profile observed under chemical etching between the *c*-plane templates and the ($11\bar{2}2$) semi-polar and ($11\bar{2}0$) non-polar templates can be explained by the change in crystal symmetry. With GaN growth occurring along the *c*-axis, the *c-* growth plane has a hexagonal symmetry. In that case, the etched nanorods possess six ($1\bar{1}00$) fairly straight m-plane facets (Figure 2a). However, when the *c*-axis is either inclined or parallel to the surface, the crystal structure on the ($11\bar{2}2$) and ($11\bar{2}0$) growth planes becomes rectangular, hence reducing the crystal symmetry. In this configuration, the ($1\bar{1}00$) m-plane facets are still present and fairly straight for the ($11\bar{2}2$) and ($11\bar{2}0$) etched nanorods (Figure 2d,g). However, the resulting change in crystal symmetry led to the formations of other facets having either different inclinations or orientations. Facets having either fewer DB per Ga atom or a preferential N-terminated surface will tend to be the most stable [23]. As a result, anisotropic crystallographic characteristics of GaN semi- and non-polar plane seriously impact the etching behaviour when the chemical component is dominant. This behaviour is particularly noticeable in the case of circular nanostructures where surfaces are created around the periphery but could be avoided for stripe patterns if aligned along <$\bar{11}23$> and <0001> for ($11\bar{2}2$) and ($11\bar{2}0$) GaN templates, respectively. Therefore, to minimize the impact of anisotropic etching behaviour occurring for semi- and non-polar plane orientations, physical dominant etching conditions should be employed.

### 3.2. Effect of Pressure

**Figure 4:**
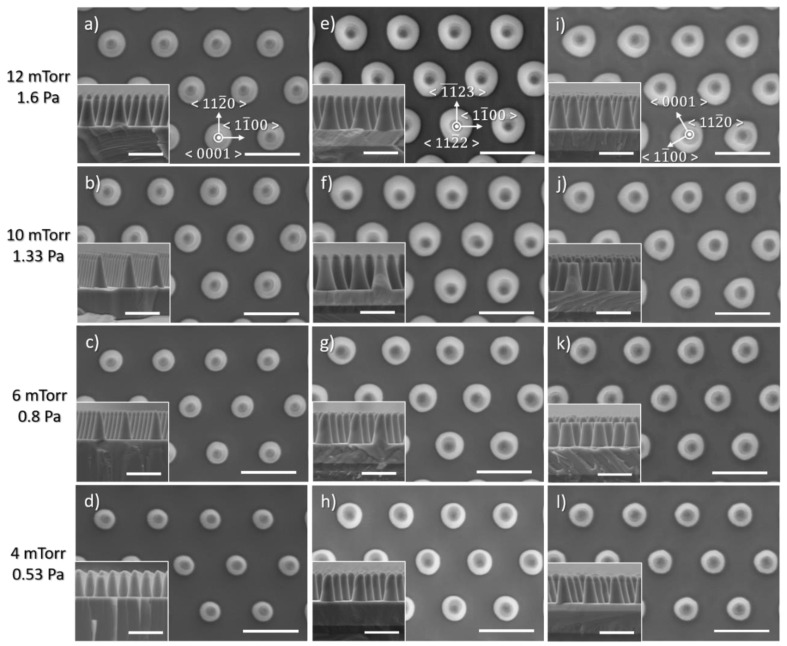
Plan view and cross-sectional (inset) SEM image of GaN nanorod etch series 2 under different chamber pressures for (**a**–**d**) *c*-plane (**e**–**h**) (11$\bar{2}2$) semi-polar and (**i**–**l**) ($11\bar{2}0$) non-polar GaN layers. Scale bar is 1 µm.

Figure 4 shows SEM images of the impact of pressure on the morphology of etched GaN nanorods. Independently of the crystal orientation, the diameter at the base of the nanorod decreases with pressure while its shape becomes clearly more circular for both semi-polar ($11\bar{2}2$) and non-polar ($11\bar{2}0$) crystal orientations, at low pressure.

**Figure 5:**
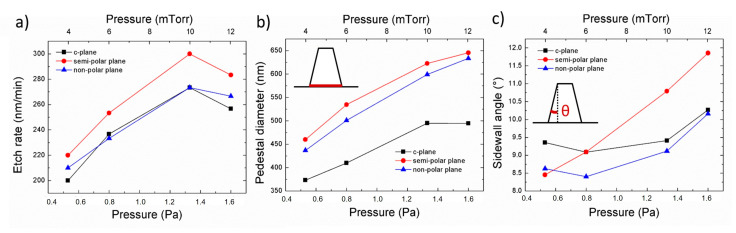
(**a**) Etch rate, (**b**) base diameter and (**c**) sidewall angle of *c*-plane, ($11\bar{2}2$) semi-polar and ($11\bar{2}0$) non-polar GaN layers as a function of the pressure. The pedestal diameter and sidewall angle are schematically described in (**b**,**c**), respectively.

Figure 5 presents the etched nanorod characteristics as a function of the pressure, for the three crystal orientations. As the pressure decreases, the etch rate, the base diameter and the sidewall angle all decrease, independently of the crystal orientation. Given that a physical dominant etching condition with 10 sccm Cl_2_ and 50 sccm Ar leads to a uniform tapering profile (constant slope of the sidewall), a decrease in the sidewall angle will reduce the pedestal diameter, and result in a lower etch depth. If we compare the nanorod diameter at a given height, its variation will follow the one of the sidewall angles.

**Figure 6:**
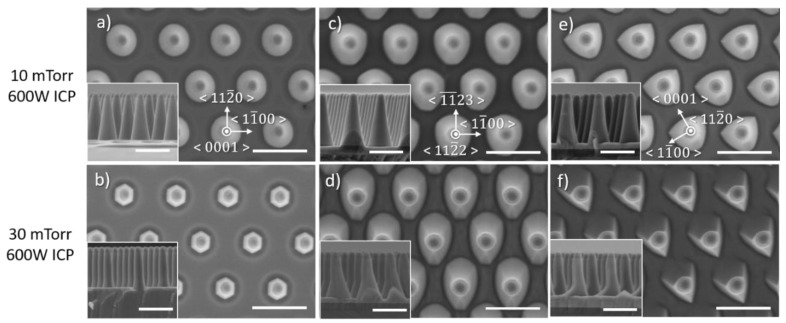
Plan view and cross-sectional (inset) SEM image of GaN nanorod etch series 3 under different chamber pressures for (**a**,**b**) *c*-plane (**c**,**d**) (11$\bar{2}2$) semi-polar and (**e**,**f**) ($11\bar{2}0$) non-polar GaN layers. Scale bar is 1 µm.

Etching experiments have been performed for both lower and higher pressures than those explored in etch series 2. However, when the other parameters of the chamber were kept fixed, the plasma was not stable due to a significant fluctuation in the RF and ICP reflected power. By tuning the ICP power to a lower value, higher pressure was achievable. Figure 6 shows the impact of high pressure under a lower ICP power. An increase from 10 mTorr (Figure 2a,c,e) to 30 mTorr (Figure 2b,d,f) results in the morphology of the nanorod being highly dependent on the crystal orientation, similar to the observations made for a Cl_2_/Ar flow rate of 50/10 and 30/30 sccm in Figure 2. Note that in this case, the sidewall angle of nanorods etched from c-plane material is almost straight at high pressure, showing the opposite trend as the one described above in Figure 4a–d and 5c. This has already been observed in the literature for c-plane etching of GaN nano and microstructure, and explained by an increased scattering and concentrations of species at high pressure that favours the formation of straight sidewall profile and even an undercut profile [23,24].

As the chamber pressure is increased, the production of the Cl-radical component increases, enhancing the chemical-etching reaction. As a result, the use of low pressure should be employed to favour physical etching and to minimize the impact of anisotropic etching occurring for semi- and non-polar plane orientations.

The change in etch rate and nanorod sidewall profile, observed independently of the crystal orientation, shows the impact of the pressure on the incident ion energy via the mean free path of species and the direct current (DC) bias, the former being high at low pressure and low at high pressure.

### 3.3. Effect of Temperature

**Figure 7:**
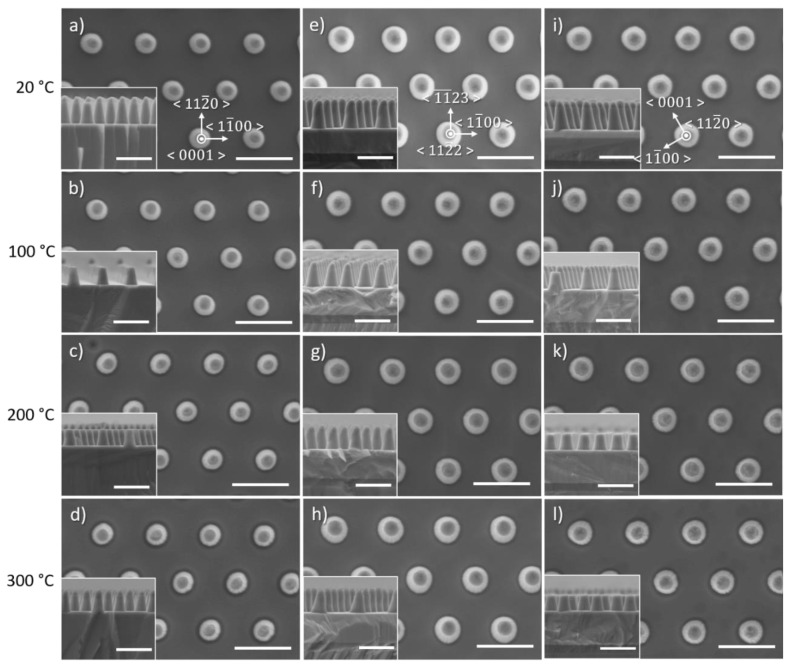
Plan view and cross-sectional (inset) SEM image of GaN nanorod etch series 4 under different chamber temperatures for (**a**–**d**) *c*-plane (**e**–**h**) (11$\bar{2}2$) semi-polar and (**i**–**l**) (11$\bar{2}0$) non-polar GaN layers. Scale bar is 1 µm.

**Figure 8:**
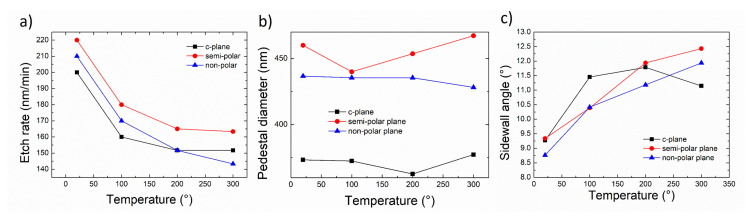
(**a**) Etch rate, (**b**) base diameter and (**c**) sidewall angle of *c*-plane, ($11\bar{2}2$) semi-polar and ($11\bar{2}0$) non-polar GaN layers as a function of temperature.

Figure 7 and Figure 8 display the effect of temperature on the morphology of etched GaN nanorods and their characteristics, respectively, for low anisotropy etching conditions (10/50 sccm Cl_2_/Ar and 4 mTorr). As the temperature increases, no noticeable change in the shape of the nanorods is observed, with the base of the nanorod being circular for all orientations. Rough sidewalls can be observed at 200 and 300 °C with the presence of striations running from the top to the bottom of the nanorods. As the etching occurs, the metal mask gradually shrinks, which can impact its outlying quality creating imperfections that can subsequently transfer to the nanorod sidewalls. In the employed conditions, a small percentage of Cl_2_ injected into the Cl_2_ + Ar plasma and a low pressure, physical etching is favoured over chemical etching with a dominant sputtering component that can significantly erode the mask. An increase in temperature in these conditions seems to further enhance the mask erosion, which could also explain the increase in the sidewall angle observed for higher temperatures (Figure 8c).

Surprisingly, the etch rate decreases with an increase of temperature, while the base diameter remains constant and the sidewall angle increases. The volatility of GaCl_x_ etched by-products is expected to be temperature dependent [25]. However, the increase of the substrate temperature could also potentially increase the desorption of Cl_2_ on the etched surface, leading to a lower etch rate [26].

Our previous reports carried out under more chemical etching conditions, i.e high percentage of Cl_2_ injected into the Cl_2_ + Ar plasma and higher pressure, revealed that higher temperatures increase the etch rate and improve the verticality of the sidewall profile of the *c*-plane etched nanorods [24,25]. We previously discussed that chemical etching conditions lead to the morphology of the nanorod being highly dependent on the crystal orientation. If these conditions were to be employed and the temperature increased, one could expect the crystal orientation to have a strong impact on the nanorod morphology.

### 3.4. Effect of RF Power

**Figure 9:**
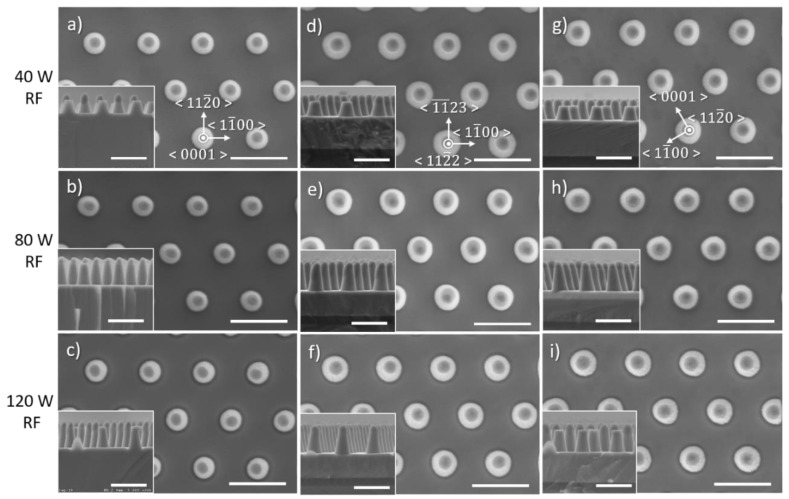
Plan view and cross-sectional (inset) SEM image of GaN nanorod etch series 5 with different radio-frequency (RF) powers for (**a**–**c**) *c*-plane (**d**–**f**) (11$\bar{2}2$) semi-polar and (**g**–**i**) (11$\bar{2}0$) non-polar GaN layers. Scale bar is 1 µm.

**Figure 10:**
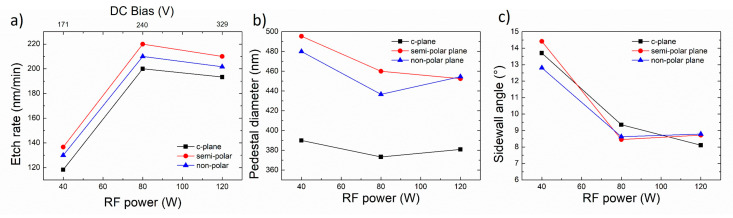
(**a**) Etch rate, (**b**) base diameter and (**c**) sidewall angle of the *c*-plane, ($11\bar{2}2$) semi-polar and ($11\bar{2}0$) non-polar GaN layers as a function of RF power.

Figure 9 and Figure 10 illustrate the impact of the RF power on the morphology of etched GaN nanorods and their characteristics, respectively. In these conditions, as the RF power decreases, the base of the nanorod remains circular and constant in size, the etch rate decreases, and the sidewall angle increases. Rough sidewalls can be observed at high RF power with the presence of striations running from the top to the bottom of the nanorod.

The change in RF power significantly impacts the DC bias (Table 1 and upper scale in Figure 10a), which is a relative measure of the kinetic energy of ions, and thus an indicator of ion penetration and sub-surface damage. Higher RF power significantly increases the DC bias, thus enhancing the physical sputtering component which can further degrade the mask. In addition, high DC bias values with high energy ions which bombard the surface have been reported to potentially generate crystal defects [27,28,29].

### 3.5. Effect of ICP Power

**Figure 11:**
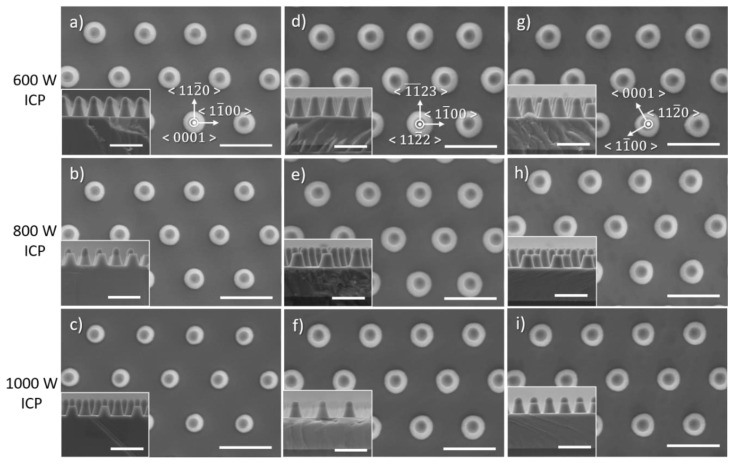
Plan view and cross-sectional (inset) SEM image of GaN nanorod etch series 6 with different inductively coupled plasma (ICP) power for (**a**–**c**) *c*-plane (**d**–**f**) (11$\bar{2}2$) semi-polar and (**g**–**i**) (11$\bar{2}0$) non-polar GaN layers. Scale bar is 1 µm.

**Figure 12:**
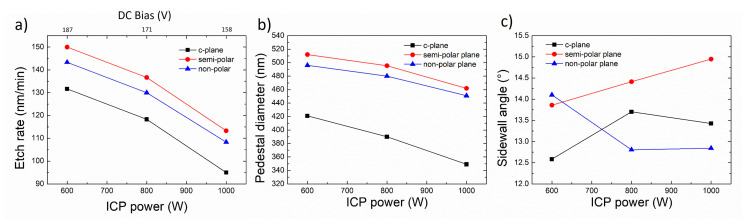
(**a**) Etch rate, (**b**) base diameter and (**c**) sidewall angle of *c*-plane. ($11\bar{2}2$) semi-polar and ($11\bar{2}0$) non-polar GaN layers as a function of ICP power.

Figure 11 and Figure 12 show the influence of the ICP power on the morphology of etched GaN nanorods and their characteristics, respectively. In these conditions, as the ICP power increases the base of the nanorod becomes more circular, reduces in size, the etch rate decreases as well as the DC bias and the sidewall angle changes. As previously discussed for the RF power, the DC bias is a relative measure of the kinetic energy of ions. As the DC bias decreases for higher ICP power, the physical sputtering component will be lowered, negatively impacting the etch rate. As the sidewall angle only shows small changes on the order of 1° as a function of the ICP power, with no similar trend observed for the various crystal orientations, the decrease in diameter observed for all crystal orientations with the decrease in ICP power mainly results from the lower etch depth.

### 3.6. Effect of Time

**Figure 13:**
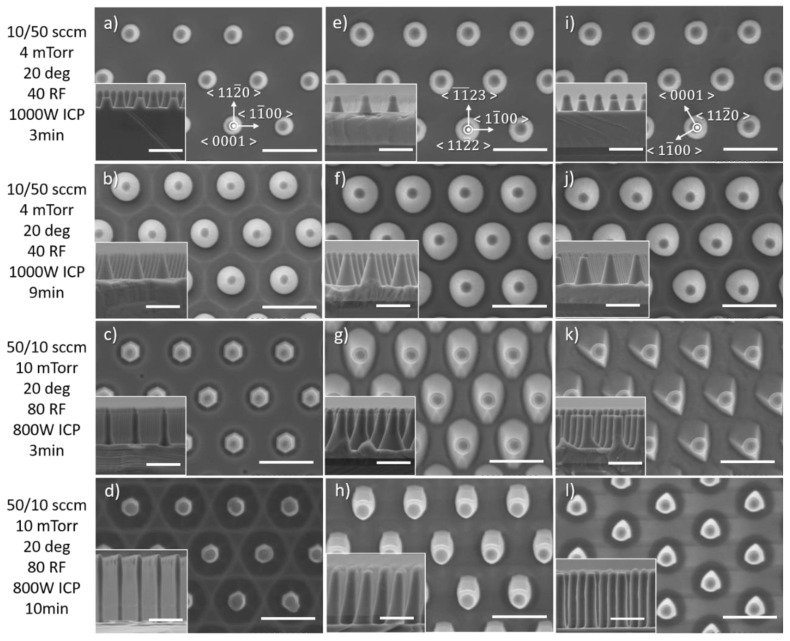
Plan view and cross-sectional (inset) SEM image of GaN nanorod etch series 7 and 8 with different times for two etching conditions, for (**a**–**d**) *c*-plane (**e**–**h**) (11$\bar{2}2$) semi-polar and (**i**–**l**) (11$\bar{2}0$) non-polar GaN layers. Scale bar is 1 µm.

The dependence on the etching duration has been performed for two etch times under two different conditions (Figure 13), either where the physical etching component or the chemical etching component is dominant. In the first case, when the etch duration is increased from 3 min (Figure 13a,e,i) to 9 min (Figure 13b,f,j) the morphology and sidewall profile of the nanorods remain similar.

The impact of the crystal orientation can however be distinguished for longer times by the distortion of the circularity of the base of the nanorod for both, semi-polar ($11\bar{2}2$) and non-polar ($11\bar{2}0$) crystal orientations. In the second case, with an increase in the etch duration from 3 min to (Figure 13c,g,k) 10 min (Figure 13d,h,l) the morphology and sidewall profile of the nanorods appear to be quite different. Most interestingly, for 10 min, the sidewall profile of the nanorod etched on semi-polar ($11\bar{2}2$) and the non-polar ($11\bar{2}0$) crystal orientations become straighter, resulting in the base of the nanorod being smaller and more circular than the one obtained after 3 min. Note also that after 10 min the sapphire substrate is reached for both the semi-polar ($11\bar{2}2$) and non-polar ($11\bar{2}0$) GaN/AlN layers. Sapphire being much harder to etch than III-nitrides, the sidewalls of the nanorods are gradually etched laterally, thus leading to a sidewall profile being more vertical at the AlN/Al_2_O_3_ interface. Such a change in sidewall profile after reaching the sapphire substrate was already observed for GaN stripe patterns aligned along the <$11\bar{2}0$> and <$1\bar{1}00$> directions [23].

## 4. Conclusions

In this paper, we investigate the impact of ICP etching conditions on the morphology, the etch rate and dimensions of GaN nanorods for various plane orientations. The parameters have been adjusted in order to minimize the impact of crystal orientation and mitigate damage to the crystal. To achieve this target, first, the chemical component was reduced by injecting a small percentage of Cl_2_ into the Cl_2_ + Ar plasma and using low pressure, and second, the DC bias was lowered by reducing the RF power and increasing the ICP power. While these conditions satisfy our targets, they appear to be incompatible with a high etch rate. This could be further improved by combining two etching conditions, a first step that provides high etch rate with an optimal neutral-to-ion ratio, and a second step where the physical component is increased to recover a regular profile and reduce the damage.

These findings have implications that go beyond the optimization of GaN nanostructures aspect ratio and shape, as the conditions can be employed to carefully design and optimize microLEDs, LEDs or other photonic structure that can be achieved from various III-nitride template orientations.

## Figures and Tables

**Table 1:** Dry etching conditions employed in the various “etch series”.

Etch Series	Cl_2_/Ar (sccm)	Pressure (mTorr)	Temperature (°C)	RF Power (W)	ICP Power (W)	Time (min)	DC Bias (V)
1	50/10–10/50	10	20	80	800	3	325–273
2	10/50	12–4	20	80	800	3	279–240
3	10/50	10–30	20	80	600	3	291–336
4	10/50	4	20–300	80	800	3	240–255
5	10/50	4	20	40–120	800	3	171–329
6	10/50	4	20	40	600–1000	3	187–158
7	10/50	4	20	40	1000	3–9	158
8	50/10	10	20	80	800	3–10	325

## Data Availability

All data created during this research is openly available from the University of Bath Research Data Archive [30].
